# Evaluation of Follow-Up CT Scans in Patients with Severe Initial Pulmonary Involvement by COVID-19

**DOI:** 10.1155/2022/6972998

**Published:** 2022-12-29

**Authors:** Behshad Pazooki, Ailar Ahangari, Mohammad-Mehdi Mehrabi Nejad, Nasim Batavani, Faeze Salahshour

**Affiliations:** ^1^Internal Medicine, Imam Khomeini Hospital Complex, Tehran University of Medical Sciences, Tehran, Iran; ^2^Tehran University of Medical Sciences, Tehran, Iran; ^3^Department of Radiology, Advanced Diagnostic and Interventional Radiology Research Center (ADIR), Imam Khomeini Hospital Complex, Tehran University of Medical Sciences, Tehran, Iran; ^4^Liver Transplantation Research Center, Imam-Khomeini Hospital, Tehran University of Medical Sciences (TUMS), Tehran, Iran

## Abstract

**Objective:**

To investigate the predictive factors of residual pulmonary opacity on midterm follow-up CT scans in patients hospitalized with COVID-19 pneumonia.

**Materials and Methods:**

This prospective study was conducted in a tertiary referral university hospital in Iran, from March 2020 to December 2020. Patients hospitalized due to novel coronavirus pneumonia with bilateral pulmonary involvement in the first CT scan were included and underwent an 8-week follow-up CT scan. Pulmonary involvement (PI) severity was assessed using a 25-scale semiquantitative scoring system. Density of opacities was recorded using the Hounsfield unit (HU).

**Results:**

The chest CT scans of 50 participants (mean age = 54.4 ± 14.2 years, 72% male) were reviewed, among whom 8 (16%) had residual findings on follow-up CT scans. The most common residual findings were faint ground-glass opacities (GGOs) (14%); fibrotic-like changes were observed in 2 (4%) patients. Demographic findings, underlying disease, and laboratory findings did not show significant association with remaining pulmonary opacities. The total PI score was significantly higher in participants with remaining parenchymal involvement (14.5 ± 6.5 versus 10.2 ± 3.7; *P*=0.02). On admission, the HU of patients with remaining opacities was significantly higher (−239.8 ± 107.6 versus −344.0 ± 157.4; *P*=0.01). Remaining pulmonary findings were more frequently detected in patients who had received antivirals, steroid pulse, or IVIG treatments (*P*=0.02, 0.02, and 0.001, respectively). Only the PI score remained statistically significant in multivariate logistic regression with 88.1% accuracy (OR = 1.2 [1.01–1.53]; *P*=0.03).

**Conclusion:**

Pulmonary opacities are more likely to persist in midterm follow-up CT scans in patients with severe initial pulmonary involvement.

## 1. Introduction

In January 2020, the World Health Organization declared COVID-19 a global pandemic. COVID-19 is highly contagious, and in severe cases, it can lead to lung involvement and acute respiratory compromise or organ failure [1]. With gradual recognition of COVID-19 pneumonia, guidelines and criteria were steadily established so as to prevent its transmission and facilitate its diagnosis and treatment [2].

Using a chest-computed tomography (CT) scan is very important for rapid diagnosis and clinical decision-making [3]. According to the World Health Organization and the Centers for Disease Control and Prevention guidelines, chest radiography and CT were major diagnostic components when COVID-19 was probable [4]. Dominant CT findings in COVID-19 pneumonia mostly include bilateral ground-glass opacities (GGOs), multifocal patchy consolidations, and peripherally distributed interstitial changes. Chest CT manifestations may differ in each patient during each stage of the disease, which can be used to differentiate a diagnosis along with the severity of pulmonary involvement (PI), follow the changes, adjust the treatment, and predict the prognosis [5].

When it comes to COVID-19 pneumonia, the recent radiology literature focuses primarily on CT findings as they are more sensitive than chest radiography [6–8]. A chest CT scan can detect small areas of GGO [9] and, therefore, is a promising imaging modality for monitoring the disease, if the radiation dose is balanced with radiologic principles. However, factors predicting long-term pulmonary sequelae have not yet been fully discussed [10]. Therefore, we designed this study to detect predisposing factors for residual pulmonary opacities on an 8-week follow-up CT scan.

## 2. Materials and Methods

### 2.1. Participants

This prospective study was reviewed and approved by the Institutional Review Board of the Ethics Committee at the Tehran University of Medical Sciences (qith reference number. IR.TUMS.VCR.REC.1399.149). Participants signed informed consent forms and were assured of the confidentiality of their data (XXXXX.REC.1399.149). The current study was conducted in a tertiary referral university hospital from March 2020 to December 2020. Inclusion criteria were as follows: (a) being hospitalized with COVID-19 pneumonia confirmed by the positive rRT-PCR assay; (b) bilateral lung involvement in the initial chest CT scan; and (c) agreement to undergo an 8-week interval follow-up CT scan. Of note, admission, discharge criteria, and treatment of all patients were based on the national protocol of COVID-19. Patients were categorized into the two following groups based on the follow-up CT scan: complete resolution and residual findings. All demographic, clinical, and paraclinical data were also compared between the two groups.

### 2.2. Data Collection

#### 2.2.1. Population Characteristics

All the following data were recorded: (a) demographic characteristics: age and sex; (b) on-admission oxygen saturation (SpO_2_, %); (c) underlying diseases: including hypertension (HTN), diabetes mellitus (DM), malignancies (solid or hematologic), or immunocompromised conditions (chemoradiation therapy and long-term corticosteroid use); (d) laboratory findings: white blood cell (1000/*μ*L), lymphocyte counts (1000/*μ*L), hemoglobin (g/dL), platelet (1000/*μ*L), erythrocyte sedimentation rate (ESR, mm/hr), and C-reactive protein (CRP, mg/L); and (e) received treatments during hospitalization: antibiotics, antiviral agents, steroid pulse, intravenous immunoglobulin (IVIG), or mechanical ventilation.

#### 2.2.2. Image Acquisition

All chest CT images were obtained at the time of admission and eight weeks later using the Siemens SOMATOM Emotion (16 slices, Erlangen, Germany) MDCT scanner. All CT scans were performed in the supine position with deep inspiration, and the imaging parameters were as follows: 5 mm slice thickness; beam collimation of 1.2 mm; tube voltage, 130 kVp; and tube current, 70 mAs.

#### 2.2.3. Image Interpretation

A board-certified radiologist with 11 years of experience in thoracic radiology was blinded to patients' outcome and interpreted chest CT images, reviewing both lung and mediastinal window settings. Chest CT scan findings were recorded according to the Fleischner Society glossary and published literature on viral pneumonia (2). The following features were considered significant remaining pulmonary findings: (a) fibrotic-like changes: traction bronchiectasis, parenchymal bands, or coarse reticular patterns; (b) faint GGO: subsegmental atelectasis, mosaic attenuation, and very faint GGO (HU <- 500) were not considered significant remaining pulmonary findings. Chest-CT-scan features included (a) predominant pattern: GGO or consolidation; (b) dominant distribution pattern: peripheral (peripheral one-third of the lung), axial, or diffuse; (c) number of involved lobes; (d) additional findings: cardiomegaly, pleural effusion, subsegmental atelectasis, parenchymal band, crazy paving, and reverse halo sign; and (f) density of opacities: using the Hounsfield unit (HU) by placing a 10 mm region of interest.

#### 2.2.4. Pulmonary Involvement (PI) Scoring System

To assess PI, a semiquantitative scoring system was proposed. All five lung lobes were visually reviewed for GGO and consolidation, and the total involvement of each lobe was scored from 0 to 5 according to the volume percentage of involvement (0: no involvement; 1: ≤5%; 2: 6–25%; 3: 26–50%; 4: 51–75%; and 5: ≥76%). The total PI score was calculated as the sum of all the five lobes' scores. The PI score ranged from 0 (no involvement) to 25 (maximum involvement). Finally, the PI density index was calculated by dividing the total PI score by the number of involved lobes.

#### 2.2.5. Statistical Analysis

We performed analyses using SPSS for Windows ver. 18 (Chicago, IL, USA). All *p* values less than 0.05 were considered statistically significant. Nominal and continuous variables were reported as the frequency (%) and mean ± standard deviation, respectively. The normality of data was evaluated by the Kolmogorov–Smirnov test. We conducted the comparisons by the independent sample *t*-test for normally distributed continuous variables; the Mann–Whitney *U* test was used for non normally distributed continuous variables, and (c) the Chi-squared test was used for nominal variables. An association between the HU and residual findings was investigated for all 250 lobes of 50 patients.

## 3. Results

Data on 50 participants (male, 36 (72%); mean age, 54.4 ± 14.2 years) were analyzed. Among those, 8 (16%) had residual findings on the follow-up CT scan. The most common residual findings were faint GGOs (14%) and fibrotic changes (4%). Thirty-two (64%) patients had at least one underlying disease. The patients' demographic, clinical, and paraclinical data in the two groups are presented in Tables 1 and 2.

Demographic data, age and sex, did not show a significant association with residual findings in CT scans. Besides, on-admission SpO_2_ in the complete resolution and residual groups was 81.5 ± 8.0 and 79.0 ± 9.7, respectively (*P*=0.46). Underlying disease was detected in 66.7% of patients in the complete resolution group and 50% in the residual group (*P*=0.25). On-admission laboratory tests also had no correlation with remaining parenchymal findings (Table 1).

Further analyses revealed that treatment regimens had significant associations with midterm chest CT findings. Remaining pulmonary opacities were more common in patients who had received antiviral, steroid pulse, or IVIG treatments (*P*=0.02, 0.02, and 0.001, respectively).

The total PI score was significantly higher in participants with remaining parenchymal involvement (14.5 ± 6.5 versus 10.2 ± 3.7; *P*=0.02). However, the total GGO, consolidation, and PI density scores showed no difference between the two groups (*P*=0.48, 0.41, and 0.14, respectively). Predominant involvement and the distribution pattern of initial compromised lung parenchyma were the same between the two groups (*P*=0.56 and 0.61, respectively). The number of involved lobes showed no association with remaining parenchymal findings (*P*=0.22). Besides, no significant difference was detected in additional findings between the two groups (Table 2).

**Figure 1:**
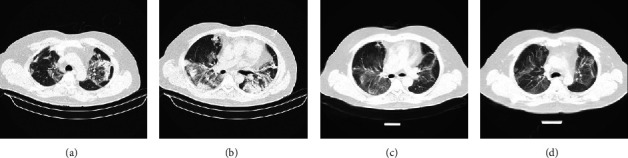
A 51-year-old man with COVID-19 pneumonia with a 17/25 severity score receiving remdesivir and dexamethasone for treatment. (a, b) The axial chest CT scan depicts air space opacities with predominant consolidation. (c, d) Chest CT 8 weeks after hospital discharge demonstrates residual faint ground-glass opacities and subsegmental atelectasis.

**Figure 2:**
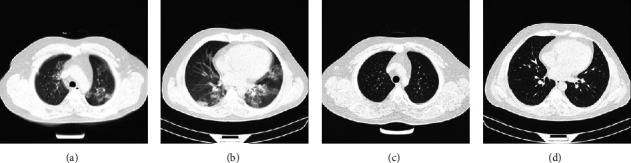
A 32-year-old man with COVID-19 pneumonia treated with oseltamivir, steroid pulse, and hydroxychloroquine. The on admission chest CT scan shows patchy ground-glass opacities (a, b) with a 10/25 severity score. The follow-up CT scan about eight weeks after hospital discharge shows no residual opacity. (c, d).

Additionally, of note, the HU of opacities in the on-admission CT scan was significantly higher in patients with remaining opacities than patients in resolved ones (−239.8 ± 107.6 in the group with residual opacities versus −344.0 ± 157.4 in resolved ones; *P*=0.01) (Figures 1 and 2).

Backward multivariate logistic regression was applied. We used residual findings as the outcome of interest and all variables with *P* value < 0.1 as independent variables. Only the total PI score remained statistically significant in multivariate logistic regression with 88.1% accuracy (OR = 1.2 [1.01–1.53]; *P*=0.03).

## 4. Discussion

We evaluated the potential predictive factors for remaining parenchymal findings in chest CT scans obtained 8 weeks after discharge. Follow-up imaging findings mainly included faint GGOs and fibrotic-like changes. We found that remaining pulmonary involvement was significantly more common in patients with a higher on-admission PI score or who received antiviral, steroid pulse, or IVIG treatments. Besides, denser opacities were more likely to persist in midterm follow-up CT scans.

GGO, GGO with consolidation, interstitial thickening, parenchymal bands, and crazy paving were the most common CT findings on midterm chest CT scans [11, 12]. A longitudinal study evaluated 51 COVID-19 patients with a median interval of 41 days after discharge. GGO, consolidation, interlobular septal thickening, irregular lines, subpleural lines, and bronchiectasis (9.8%, 1%, 35.3%, 15.7%, 7.8%, and 3.9%, respectively) were found in follow-up chest CT scans which were significantly improved compared to those right before discharge [13].

Factors predicting the midterm chest CT findings were investigated through limited previous studies. The largest study investigated the predictive factors of parenchymal fibrotic changes in a six-month CT scan of 114 patients. Fibrotic changes were detected in 35% of patients. A higher initial PI score was found in patients with remaining parenchymal involvements, which aligned with our findings. [14]. Similar results were also reported in another study of 41 patients in a seven-month follow-up investigation [15]. Another study evaluating the three-month follow-up CT scans of 52 COVID-19 patients reported GGOs (54.5%) and subpleural parenchymal bands (31.8%) as the most common findings on midterm images. Also, a higher on-admission PI score was found in patients with residual findings [12]. Another retrospective study evaluated 14 patients with fibrosis and 18 patients without fibrosis on the follow-up CT scan. It was found that patients with older age and higher levels of inflammatory indicators (CRP and interleukin-6) end up more frequently in the fibrotic group. Longer hospital stay, pulsed steroid therapy, and antiviral therapy were also associated with fibrosis [11]. A prospective study on 80 participants showed 48% GGOs (48%), bands (37%), and fibrosis (12%) in COVID-19 patients after about 3 months. The predictors of midterm sequelae in this study were levels of CRP, fibrinogen, urea, and creatinine, age, hospital stay, and mechanical ventilation [16].

The PI score has been confirmed to predict the short-term outcome and prognosis in COVID-19 patients [8]. In this study, we found that patients with extended parenchymal involvement are more likely to show residual findings in follow-up images. Therefore, they may benefit from follow-up CT examinations due to some extent of pulmonary sequelae. However, studies with long-term follow-up are required to confirm our results.

Our further analyses showed higher rates of residual findings in patients who had received antiviral, steroid pulse, or IVIG treatments during hospitalization. Antibiotic therapy and mechanical ventilation were not significantly associated with residual findings. Yu et al. also found a significant association between pulmonary sequelae and steroid pulse and antiviral treatments in 32 COVID-19 patients [11]. Another study also found a higher probability of pulmonary fibrotic changes in patients who underwent steroid therapy or mechanical ventilation in a seven-month CT scan [15]. However, only steroid therapy remained statistically significant in multivariate logistic regression, and the PI score was the only independent predictive variable in our multivariate logistic regression. We assume that since patients with more severe initial pulmonary involvement undergo more intensive treatments, the relationship between these treatments and residual findings might be biased. Altogether, we believe that extent of initial PI is the best predictor of midterm pulmonary findings.

Our study has several limitations. We only included hospitalized patients that may not properly reflect the midterm sequelae of COVID-19 in the community. The subjective nature of qualitative and semiquantitative chest CT scan evaluations can result in different imaging findings and should be replaced with more accurate quantitative measurements in later studies. Future studies on larger populations and long-term imaging follow-ups of COVID-19 patients could reveal the potential burden of this disease on the involved organs including the lungs, which changes further approach to therapeutic strategies of COVID-19. We also recommend spirometry measurements to evaluate the long-term consequences of novel coronavirus pneumonia in the future. However, sufficient evidence of chronic complications and remaining pulmonary sequelae in COVID-19 patients is still lacking due to the novelty of this virus and diversity in its findings.

In conclusion, we found significantly higher residual pulmonary parenchymal involvement in patients with higher initial PI scores and those receiving antiviral therapy, steroid pulse, or IVIG treatments. Besides, opacities with higher HUs were more likely to persist in midterm imaging. We recommend that patients with severe initial pulmonary involvement should receive intensive care and be followed to minimize further sequelae.

## Figures and Tables

**Table 1:** Details of demographic and clinical data of patients and differences between the two groups.

Variables	Patients	Complete resolution	With residual findings	*P*‐value
	*N* = 50	*N* = 42	*N* = 8	
Demographic data				
Age^*∗*^	54.4 (14.2)	53.6 (14.6)	58.7 (10.9)	**0.39**
Gender				
Male	36 (72%)	30 (71.4%)	6 (75%)	**0.84**
Female	14 (28%)	12 (28.6%)	2 (25%)	

Clinical data				
SpO_2_^*∗*^	81.1 (8.2)	81.5 (8.0)	79.0 (9.7)	**0.46**
Underlying disease	32 (64%)	28 (66.7%)	4 (50%)	**0.25**
Laboratory findings^*∗*^				
WBC	9.4 (3.8)	9.3 (3.5)	10.1 (3.1)	**0.57**
Lymphocyte	2.2 (2.6)	2.4 (2.7)	1.3 (1.1)	**0.33**
Hemoglobin	12.5 (2.5)	12.4 (2.6)	12.8 (1.6)	**0.72**
Platelet	176.0 (95.2)	166.8 (98.9)	229.6 (41.8)	**0.11**
ESR	63.9 (38.3)	63.1 (36.0)	68.4 (52.1)	**0.74**
CRP	119.9 (76.2)	113.4 (77.5)	155.4 (61.2)	**0.18**
Treatment				
Antibiotic	24 (48)	21 (50)	3 (37.5)	**0.52**
Antiviral	32 (64)	24 (57.1)	8 (100)	**0.02**
Steroid pulse	9 (18)	5 (11.9)	4 (50)	**0.01**
IVIG	2 (4)	0 (0)	2 (25)	**0.001**
Mechanical ventilation	20 (40)	16 (38.1)	4 (50)	**0.53**

^*∗*^mean (standard deviation); all other variables were reported as *N* (%). WBC = white blood cell; ESR: erythrocyte sedimentation rate; CRP = C-reactive protein; IVIG: intravenous immunoglobulin.

**Table 2:** Radiologic findings in all patients and differences between the two groups.

Variables	Patients	Complete resolution	With residual findings	*P*‐value
	*N* = 50	*N* = 42	*N* = 8	
PI scores^*∗*^				
Total GGO score	8.5 (4.6)	8.4 (4.2)	9.7 (6.5)	**0.48**
Total consolidation score	3.5 (3.9)	3.2 (3.5)	5.1 (5.8)	**0.41**
Total PI score	10.8 (4.3)	10.2 (3.7)	14.5 (6.5)	**0.02**
PI density index	3.1 (0.7)	3.1 (0.7)	3.5 (0.7)	**0.14**

Predominant pattern				
GGO	40 (80)	33 (78.6)	7 (87.5)	**0.56**
Consolidation	10 (20)	9 (21.4)	1 (12.5)	

Dominant distribution of lesions				
Peripheral	26 (52)	23 (54.8)	3 (37.5)	**0.61**
Axial	22 (44)	17 (40.5)	5 (62.5)	
Diffuse	2 (4)	2 (4.8)	0 (0)	

Involved lobes	3.6 (1.4)	3.4 (1.4)	4.1 (1.3)	**0.22**

Additional findings				
Cardiomegaly	19 (38)	15 (35.7)	4 (50)	**0.69**
Pleural effusion	6 (12)	6 (14.3)	0 (0)	**0.25**
Subsegmental atelectasis	29 (58)	26 (61.9)	3 (37.5)	**0.20**
Parenchymal band	21 (42)	17 (40.5)	4 (50)	**0.62**
Crazy paving	14 (28)	11 (26.6)	3 (37.5)	**0.51**
Reverse halo	6 (12)	5 (11.9)	1 (16)	**0.96**

^*∗*^mean (standard deviation); all other variables were reported as *N* (%). PI = pulmonary involvement; GGO = ground-glass opacity.

## Data Availability

The data used to support the findings of this study are available from the corresponding author upon request.
